# Statin Improves Flow-Mediated Vasodilation in Chronic Kidney Diseases

**DOI:** 10.1155/2013/876865

**Published:** 2013-12-11

**Authors:** Tsuneo Takenaka, Hiroshi Takane, Tomohiro Kikuta, Yusuke Watanabe, Hiromichi Suzuki

**Affiliations:** ^1^International University of Health and Welfare, Clinical Research Center, Sanno Hospital, 8-10-16 Akasaka, Minato, Tokyo 107-0052, Japan; ^2^Saitama Medical University, Department of Nephrology, 36 Morohongo, Moroyama, Iruma, Saitama 350-0495, Japan

## Abstract

*Background*. Numbers of drugs are required to manage patients with chronic kidney disease (CKD). Drug adherence is relatively poor in this population. *Methods*. In 36 CKD patients with hypertension and dyslipidemia, who were prescribing amlodipine 5 mg and atorvastatin 10 mg daily, the influences of exchanging to a combination drug containing equivalent doses of amlodipine and atorvastatin were observed for 6 months. *Results*. At the baseline, flow-mediated dilation (FMD) was reduced (2.4 ± 0.3%), and proteinuria was significantly contributed to decrements of FMD (*R* ^2^ = 0.38, *F* = 3.7, df (6,29), and  *P* < 0.01). Six months later from exchanging to combination drug, total cholesterol (TC, 197 ± 5 to 183 ± 3 mg/dL,  *P* < 0.01) and triglycerides (142 ± 14 to 129 ± 10 mg/dL, *P* < 0.05) were decreased, but high density lipoprotein cholesterol (53 ± 3 to 56 ± 3 mg/dL, *P* < 0.05) was increased. FMD was slightly albeit significantly improved to 2.7 ± 0.3% (*P* < 0.05). No serious adverse effects were seen by the combination drug. Subanalysis for the patients with considerable reductions of TC demonstrated that the combination drug decreased proteinuria and high sensitive CRP (*P* < 0.05 for both). *Conclusion*. Our data indicate that proteinuria constitutes a determinant of a reduced FMD. The present results implicate that combination drug is useful to improve adherence and suggest that atorvastatin refines endothelium function as well as lipid profiles in CKD patients.

## 1. Introduction

Patients with chronic kidney diseases (CKD) are at high cardiovascular risk [1]. Accelerated atherosclerosis prevails in this patient population [2]. Hypertension is one of cardiovascular risk factors in patients with CKD. High blood pressure elicits endothelial injury directly and indirectly [3]. Atherosclerosis is an inflammatory disease triggered by endothelial dysfunction, which leads to cholesterol flux into the arterial wall. Macrophages in the vessel wall uptake denatured low density lipoprotein cholesterol (LDL-C), forming foam cells and generating the typical atherosclerotic lesions [4]. In addition to traditional cardiovascular risks, CKD patients are exposed to other risks including abnormal calcium-phosphate metabolism, decreased synthesis of nitric oxide (NO), and excessive oxidative stress [5, 6]. It is important to maintain the endothelium intact to prevent the development and progression of atherosclerosis.

CKD patients frequently manifest dyslipidemia, such as hypercholesterolemia, as well as hypertension. Proteinuria could be linked to an increase in cholesterol, as evident in nephrotic syndrome [7]. To control the above-mentioned cardiovascular risk factors, multiple drugs are required to manage CKD patients. However, drug adherence is erratic in this population [8]. Recently, combination drugs have become available. Many pills containing an angiotensin receptor blocker plus a diuretic or calcium channel blocker are prescribed. Caduet is a unique drug that contains both an antihypertensive and lipid-lowering agent, presumably suited for hypertensive patients with dyslipidemia [9]. The usefulness of the latter combination drug for CKD patients was assessed in this study.

Endothelial function can be examined by various methods [10–12], including counting endothelial progenitor cells and assessing acetylcholine dilation and flow-mediated dilation (FMD). FMD is a noninvasive method to evaluate endothelium-derived NO bioavailability on the arterial wall. Like virtually all tests, it has a weakness that it does not reflect the exact synthesis of NO from the endothelium but does provide information on the circumstances surrounding the endothelium such as a diabetic and uremic milieu [10, 13]. Although proteinuria is a strong cardiovascular risk factor [1], the relation of proteinuria with endothelial function has not been adequately characterized in hypertensive CKD patients. Our working hypothesis is as follows [14]: the proximal tubule uptakes protein leaked from glomeruli and generates oxidative stress to break down protein of glomerular origin. The oxidative stress can spread from the kidney to the systemic circulation, eliciting endothelial dysfunction and vascular inflammation. Statins may lower tubular stress [15]. Our study data indicated that a combination drug is effective for CKD patients to improve adherence. Furthermore, our results suggested that atorvastatin had beneficial effects on not only the lipid profile but also endothelial function in CKD patients.

## 2. Methods

This is a retrospective cohort-based study that utilized medical records from April 2011 to March 2012 on patients who visited our outpatient facility for management of CKD. All patients had consented for use of their clinical data for research purposes. Subjects were 36 CKD patients with hypertension and dyslipidemia who were taking amlodipine 5 mg and atorvastatin 10 mg daily in the morning. From their clinical data, we determined the effects of changing the administration of these two drugs to a combination drug (Caduet) containing equivalent doses of amlodipine and atorvastatin after a 6-month period. In these patients, central blood pressure was estimated by radial artery tonometry, and FMD was measured using echography on the brachial artery. At the time of entry, some patients were also taking other antihypertensives including angiotensin receptor blockers, angiotensin converting enzyme inhibitors, direct renin inhibitor, beta-blockers, alpha-blockers, diuretics, and drugs acting on central nervous system (CNS). We did not exclude such patients, but dosages of the other medications were not changed during observation periods [16].

All of the subjects had switched their once-daily amlodipine and atorvastatin to a combination drug. No washout period was set due to ethical considerations. The office systolic blood pressure (SBP) and diastolic blood pressure (DBP) were measured after the patient had rested for at least 5 min between 9 and 11 AM using a mercury sphygmomanometer (the first and the fifth Korotkoff sounds were used to identify the SBP and DBP, resp.). Two measurements were done after the patient had been in the sitting position for 5 and 10 min, respectively, and the average of the 2 values was defined as the blood pressure for efficacy analysis. The target blood pressure was ≤130/80 mmHg. Amlodipine was taken once in the morning. If BP was not controlled, the dose of amlodipine would have been titrated, but this was not required. Patients were requested to continue their usual diet and daily activities during the study period. We had only analyzed data for 6 months because a longer study period might have obscured the effects of drug exchange by the alteration of other factors such as diet and daily activities [17].

Exclusion criteria were as follows: a history of recent (<6 months) myocardial infarction, unstable angina, heart failure, and cerebrovascular events; severe hepatic disease; CKD stage 5; and a history of malignancy. The criteria for discontinuing the study were uncontrolled hypertension (>170/110 mmHg), persistent hypotension (SBP < 110 mmHg), doubling of serum creatinine, and the requirement for renal replacement therapy. The study was conducted according to Good Clinical Practice and the Declaration of Helsinki [16].

At the time of exchange, as well as 6 months later, FMD, central and brachial blood pressures, laboratory data including urinary protein, serum creatinine and high sensitive C-reactive protein (CRP) were measured while fasting. The estimated GFR (eGFR) was calculated by using the MDRD equation modified for Japan [18]. Radial artery pressure pulse waveforms were recorded with an automated tonometric system (HEM-9000AI; Omron Healthcare, Kyoto, Japan) while the patient was in the sitting position [16]. The waveform was calibrated automatically using a built-in oscillometric brachial sphygmomanometer. The algorithm of HEM-9000AI automatically performed online detection of the second peak (late systolic inflection) based on the second maximum of the fourth derivative of the radial pressure waveform to determine the late or second SBP (SBP2) as an index of central blood pressure. Two measurements were obtained 1 min apart and averaged for analysis. FMD was assessed by echography on the brachial artery (UNEX-EF, UNEX Co. Ltd., Nagoya, Japan). At first, baseline measurement of the arterial diameter was performed and continuously measured at the same position. Then, the brachial artery was occluded by the cuff with pressure higher than SBP for 5 min to stop the blood flow. Brachial arterial diameter was also recorded after the release of the cuff. FMD was assessed when maximal dilation was observed and expressed as percent changes from the baseline [10]. A single observer performed all arterial function measurements to avoid interobserver errors.

Results are expressed as the mean ± SEM. Student's  *t*-test was employed for comparisons. Simple or multiple regression analysis was also applied. Statistical significance was defined as  *P* < 0.05. All analyses were performed with SPSS software (SPSS, Inc. Chicago, IL, USA, Ver. 17).

## 3. Results

Patient data are shown in Table 1. Blood pressure was fairly well controlled, and the average eGFR was 39 ± 17 mL/min. In addition to amlodipine, most patients were taking renin angiotensin system (RAS) inhibitors, but no patient was taking two RAS inhibitors (i.e., angiotensin receptor blocker plus converting enzyme inhibitor, etc.) simultaneously. Diuretics were prescribed for 20 patients, and alpha or beta blockers were prescribed for several patients. Only a few patients were taking CNS-acting antihypertensive medications. Kidney diseases among 29 of the study patients were diabetic nephropathy [11], nephrosclerosis [8], IgA nephropathy [7], membranous nephropathy [2], and polycystic kidney disease [1]. The underlying renal diseases in the remaining 7 patients were not well characterized. Four patients were at CKD stage 2, 11 at stage 4, and 21 at stage 3. In the CKD patients, FMD was markedly reduced to 2.3 ± 0.2% compared to the control value (>6%).

Firstly, univariate regression analysis of FMD was performed using as independent variables various patient data for which we had information in order to explore the factors that contributed to a reduction in FMD (Table 2). Serum creatinine instead of the eGFR was applied as an independent variable because age was already used to calculate the eGFR. Diabetes and urinary protein significantly reduced FMD in these patients. Secondly, multivariate regression analysis against FMD was performed using variables with  *P* < 0.2  in univariate regression as independent variables. Among three blood pressure parameters, SBP was used to reduce multicollinearity with SBP2. As shown in Table 3, urinary protein was selected as the factor that contributed to a reduction in FMD (*R* ^2^ = 0.38,  *F* = 3.7, df (6,29), and *P* < 0.01).

**Figure 1:**
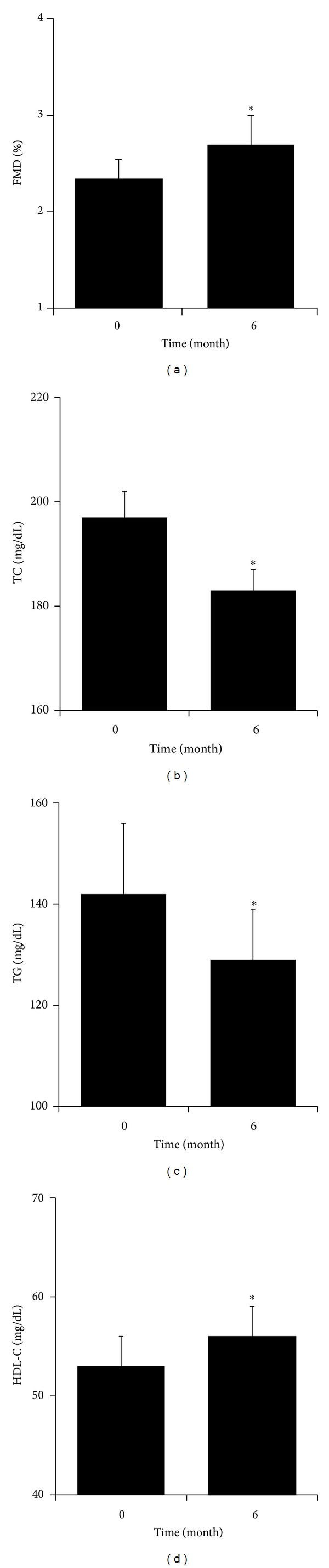
Temporal changes in flow-mediated dilation (a) and lipid profiles ((b)–(d)). FMD: flow-mediated dilation; TC: total cholesterol; TG: triglycerides; and HDL-C: high density lipoprotein cholesterol. *Significant difference from baseline.

Both systolic and diastolic blood pressures (to 135 ± 2/74 ± 1 mmHg), including central blood pressure (to 122 ± 3 mmHg), and pulse rate (to 74 ± 2 bpm) did not alter during the observation period. Proteinuria (to 0.96 ± 0.11 g/gCr) and serum creatinine (to 1.44 ± 0.08 mg/dL) remained unchanged. However, FMD was slightly, albeit significantly, (*P* < 0.05) increased at 6 months from the change to a combination drug (Figure 1). In addition, lipid profiles were improved after the change to a combination drug (Figure 1). Total cholesterol (TC, *P* < 0.01) and triglycerides (TG, *P* < 0.05) were reduced, whereas high density lipoprotein cholesterol (HDL-C) was elevated (*P* < 0.05). No serious adverse effects such as rhabdomyolysis or liver dysfunction were observed in those using the combination drug. Indeed, neither high sensitive CRP (to 1.1 ± 0.1 mg/L) nor creatine kinase (149 ± 16 to 152 ± 17 U/L) was altered by the switch in medication.

**Figure 2:**
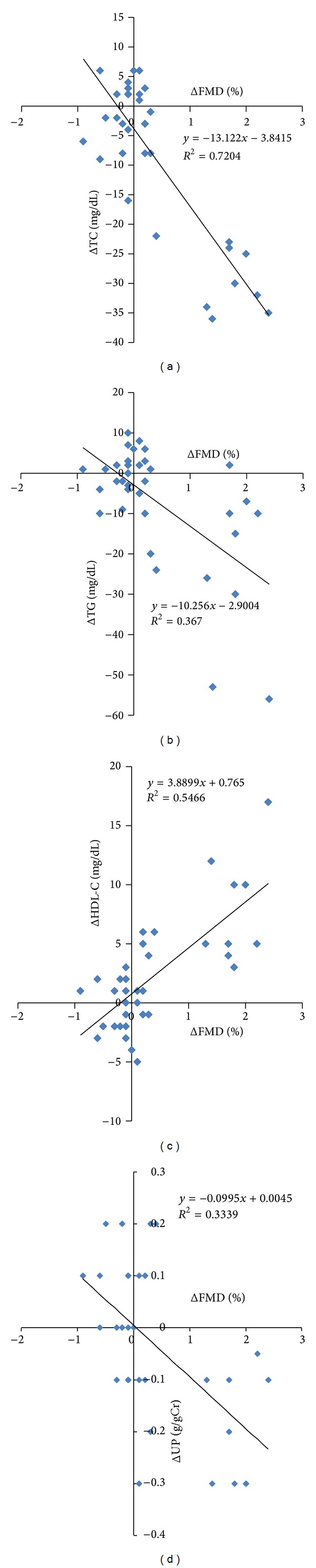
Relationship between changes in flow-mediated vasodilation (FMD) and that of TC (*P* < 0.0001), TG (*P* < 0.0001), HDL-C (*P* < 0.0001), and UP (*P* < 0.05). TC: total cholesterol; TG: triglycerides; HDL-C: high density lipoprotein cholesterol; and UP: urinary protein.

Since FMD as well as lipids was altered by the use of the combination drug, univariate regression was performed for changes in FMD using changes in lipid parameters as the independent variable. Changes in three lipid parameters correlated with that of FMD (Figure 2). Changes in non-HDL cholesterol also correlated with those of FMD (slope: −0.043 g/gCr/%, *R* ^2^:  0.72, *P* < 0.0001). Surprisingly, there was a significant correlation between changes in FMD and those of proteinuria (Figure 2). Next, multivariate regression for changes in FMD was performed using lipid data and urinary protein as independent variables (Table 4). The analysis indicated that changes in TC and urinary protein participated in changes in FMD (*R* ^2^ = 0.81,  *F* = 32, df (4,31), and  *P* < 0.0001).

A subanalysis was performed on 11 patients whose TC was decreased more than 10 mg/dL by the switch to the combination drug because these patients seemed not to adhere to the original medications and because a separate analysis would better characterize the effects of the combination drug. The subanalysis revealed that the switch to a combination drug decreased proteinuria (1.25 ± 0.28 to 1.12 ± 0.24 g/gCr, *P* < 0.05) and high sensitive CRP (1.0 ± 0.2 to 0.7 ± 0.1 mg/L, *P* < 0.05), in addition to the trends similar to the mother data (reductions in TC and TG, and increments in FMD and HDL-C without significant changes in the other parameters including blood pressure or eGFR).

## 4. Discussion

Clinical trials have demonstrated that antihypertensive drugs improve cardiovascular and renal prognosis in hypertensive and CKD patients [19, 20]. However, clinical trials with lipid lowering agents have to provide consistent results. Data from the Collaborative Atorvastatin Diabetes Study (CARDS) showed that atorvastatin reduced major cardiovascular events and preserved eGFR in patients with diabetic nephropathy [21]. The Treating to New Targets (TNT) study showed that eGFR was increased by large-dose atorvastatin in patients with coronary heart disease and CKD [22]. In accordance, the Study of Heart and Renal Protection (SHARP) trial reported that lipid lowering with simvastatin plus ezetimibe decreased cardiovascular events in CKD patients [23]. However, the Study to Evaluate the Use of Rosuvastatin in Subjects on Regular Haemodialysis: an Assessment of Survival and Cardiovascular Events (the AURORA study) showed that rosuvastatin did not suppress cardiovascular death, nonfatal myocardial infarction, or nonfatal stroke in hemodialysis patients [24]. Results of these studies suggested that differing CKD stages could confound the effects of statins. Thus, the present study did not include patients at CKD stage 5.

Chang et al. reported that adherence to both antihypertensive drugs and statins decreased to 50% in the subsequent 3 years in CKD patients [8]. In contrast, the present findings showing that a switch to a combination drug improved lipid profiles without changes in blood pressure suggested that adherence to the statin had been worse than adherence to an antihypertensive drug. We have recommended that all of our patients measure blood pressure at home. About a half agreed with this policy and monitor home blood pressure twice a day. Thus, they can learn if their blood pressure rises when they discontinue antihypertensive drugs. Results of questionnaires administered to patients support the above considerations (not shown). This might account for the differences between our findings and those of Chang et al. in that home blood pressure monitoring would provide a motivation to adhere to antihypertensive drugs differing from that of statins.

The present data indicated that FMD was prone to be reduced in the CKD patients, suggesting that endothelial NO synthesis is decreased in this population. Prolonged hypertension in CKD elicits endothelial cell damage [3]. There are increases in asymmetric dimethyl-arginine, an endogenous inhibitor of NO synthesis, in CKD patients [6]. Indoxyl sulfate is accumulated in the CKD milieu and provides potent oxidative stress [10]. Reactive oxygen species break down NO and weaken the action of NO. Glucose itself modulates NO action [13]. Our results constitute the first demonstration that proteinuria is a contributing factor to a reduced endothelial function. Proteinuria induces oxidative stress as one of causes that suppress FMD. Proteins filtered from glomeruli are up-taken by proximal tubular cells, thereby eliciting oxidative stress to break down proteins [14–16]. Oxidative stress should spread from the kidney to the systemic circulation via oxidized particles such as LDL-C [25], presumably inducing further endothelial dysfunction and atherosclerosis.

**Figure 3:**
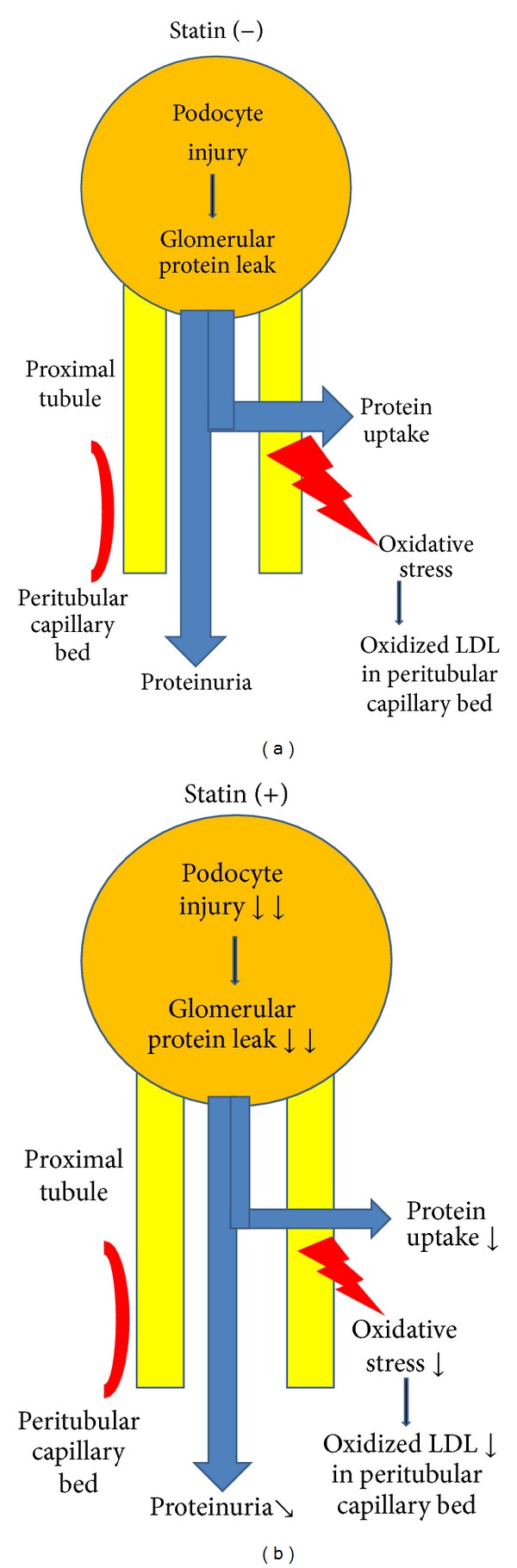
Multiple actions of statin on chronic kidney diseases: statin decreases proximal tubular uptake of protein leaked from glomeruli, reducing oxidative stress. Statin also improves podocyte injury, reducing glomerular protein leak. To obtain significant reductions in proteinuria by statin, the decrements in glomerular protein leak must be larger than those of proximal tubular uptake of protein by statin.

Previous findings indicated that atorvastatin reduced proteinuria in nondiabetic CKD patients and that it improved renal function and podocyte injury in diabetic nephropathy [26, 27]. Subanalysis in the present data revealed that atorvastatin decreased protein excretion. Mason et al. reported that atorvastatin enters deep inside of the cell membrane to help calcium channel blocking [28]. Sympatholytic action of the statin may be involved [29]. Podocytes have TRP6 channels that share approximately 70% molecular similarity to L-type calcium channels [30, 31]. An increase in calcium flux through TRP6 induces podocyte injury and proteinuria. Regardless of the precise mechanisms of how statin protects the kidney, these observations suggest that atorvastatin is renoprotective. However, the degree of reduction in proteinuria was too small to fully account for an improvement in FMD by the combination drug in accordance with a relationship between proteinuria and FMD (Tables 2 and 3). Of interest, statin inhibits protein uptake by proximal tubules [15], reducing tubular stress and generation of oxidative stress from the kidney more potently than changes in proteinuria (Figure 3).

As stated, CKD is characterized by excessive oxidative stress, which induces arterial contraction and remodeling [14, 16, 25]. Atorvastatin possesses pleotropic actions such as potent anti-inflammatory and antioxidant effects, suggesting that it improves FMD acutely by increasing the bio-availability of vasodilatory NO and chronically by altering vascular remodeling including an increase in endothelial NO synthase expression [12, 32]. Of interest, Zwaka et al. demonstrated that macrophages uptake native LDL in the presence of CRP to form foam cells [33]. The JUPITER study (Justification for the Use of Statins in Primary Prevention: An Intervention Trial Evaluating Rosuvastatin) showed that rosuvastatin decreased high sensitivity CRP [34]. However, fluvastatin failed to alter high sensitive CRP in hypertensive patients with normocholesterolemia [35]. Although the precise reasons for the discrepancies are not readily apparent, they may relate to the differences in methodology between studies. There are differences according to the specific statin used. Ozaki et al. reported that 10 mg of atorvastatin decreased high sensitivity CRP in hypercholesterolemia [36]. Although the present mother data failed to confirm a reduction in CRP by switching to a combination drug, the subanalysis demonstrated that atorvastatin decreased high sensitive CRP in CKD patients. Collectively, these data suggest that statin suppresses vascular microinflammation and oxidative stress, thereby improving endothelial function, and imply that a combination drug is useful for CKD patients to improve their adherence and vascular health.

The present study had several limitations. Because this retrospective study examined data on patients from a single center in one country, there is a potential bias regarding patient selection. Although statistical significance was attained, the sample size was relatively small especially for the subanalysis. Only the active treatment group was examined. Larger randomized controlled studies across a number of countries will be required to draw final conclusions. In addition, oxidized LDL-C was shown to correlate with total LDL-C [25]. Changes in TC were related to those of FMD in the present study (Table 4). However, TC was not selected as a significant confounding factor to FMD at baseline (Table 2). Taken together, the present data failed to dissect the effects of LDL lowering on FMD out of the pleotropic actions of atorvastatin [37]. Thus, great caution is required when generalizing the results. However, our results support the safety of the combination drug. Furthermore, the present data are consistent with previous studies [21, 22, 34] and suggest that atorvastatin is effective for improving endothelial function in CKD patients.

In summary, the present data provided evidence that the combination drug is safe and effective for CKD patients to improve adherence and lipid profiles. In addition, our results indicate that proteinuria is involved in the reduced endothelial function in CKD. Finally, the present findings suggest that pleotropic actions of atorvastatin including decreasing proteinuria and high sensitive CRP participate in an improvement of FMD by a combination drug in CKD patients.

## Figures and Tables

**Table 1:** Data on study patients.

	Baseline	Caduet
Age (y)	65 ± 2	
Sex (male: %)	52	
Diabetes (%)	31	
Serum creatinine (mg/dL)	1.43 ± 0.07	1.44 ± 0.08
Urinary protein (g/gCr)	0.99 ± 0.12	0.96 ± 0.11
Blood pressure (mmHg)	133 ± 2/74 ± 1	135 ± 2/74 ± 1
Pulse rate (bpm)	75 ± 2	74 ± 2
Total cholesterol (mg/dL)	196 ± 3	187 ± 2*
Triglycerides (mg/dL)	138 ± 8	131 ± 7*
HDL-C (mg/dL)	52 ± 2	55 ± 2*
High sensitive CRP (mg/L)	1.2 ± 0.1	1.1 ± 0.1
Central blood pressure (mmHg)	124 ± 3	122 ± 3
Flow-mediated dilation (%)	2.3 ± 0.2	2.7 ± 0.2*

Medications		

Angiotensin receptor blockers	17 patients	
Direct renin inhibitors	12 patients	
Converting enzyme inhibitor	4 patients	
Diuretics	20 patients	
Beta blockers	8 patients	
Alpha blockers	7 patients	
CNS-acting antihypertensives	3 patients	

Cr: creatinine; bpm: beats per minute; CRP: C-reactive protein; and HDL-C: high density lipoprotein cholesterol. *Indicates significant difference from respective baseline value.

**Table 2:** Simple regression to flow-mediated dilation.

	Slope	*t*	*P*
Age (y)	−0.02	−1.71	0.1
Sex (M = 0/F = 1)	0.06	0.14	0.88
Diabetes	−1.04	−2.57	0.01
SCr (mg/dL)	0.08	0.17	0.86
UP (g/gCr)	−0.75	−3.25	0.005
SBP (mmHg)	−0.03	−1.54	0.13
DBP (mmHg)	−0.04	−1.23	0.22
PR (bpm)	−0.01	−0.14	0.88
TC (mg/dL)	−0.01	−0.62	0.54
TG (mg/dL)	0.01	1.88	0.07
HDL-C (mg/dL)	0.02	1.41	0.16
SBP2 (mmHg)	−0.02	−1.51	0.15
CRP (mg/dL)	−0.23	−0.67	0.50

SCr: serum creatinine; UP: urinary protein; SBP: systolic blood pressure; DBP: diastolic blood pressure; PR: pulse rate; TC: total cholesterol; TG: triglycerides; HDL-C: high density lipoprotein cholesterol; and CRP: C-reactive protein. Presence/absence of diabetes was counted as 1/0.

**Table 3:** Multivariate regression to flow-mediated vasodilation.

	*β*	*t*	*P*
Age (y)	−0.02	−0.9	0.39
Diabetes	0.23	0.79	0.44
UP (g/gCr)	−0.69	−2.74	0.01
SBP (mmHg)	−0.02	−0.94	0.35
TG (mg/dL)	0.01	0.06	0.95
HDL-C (mg/dL)	0.51	0.2	0.84

UP: urinary protein; SBP: systolic blood pressure; TG: triglycerides; and HDL-C: high density lipoprotein cholesterol. Presence/absence of diabetes was counted as 1/0.

**Table 4:** Multivariate regression to the change in flow-mediated dilation.

	*β*	*t*	*P*
ΔTC	−0.05	−4.77	0.0001
ΔHDL-C	0.04	1.65	0.11
ΔTG	0.01	1.59	0.12
ΔUP	−1.56	−3.09	0.01

TC: total cholesterol; TG: triglycerides; HDL-C: high density lipoprotein cholesterol; UP: urinary protein. Non-HDL cholesterol was excluded from the independent variables to reduce multicollinearity.

## References

[1] Sarnak MJ, Levey AS, Schoolwerth AC (2003). Kidney disease as a risk factor for development of cardiovascular disease: a statement from the American Heart Association Councils on kidney in cardiovascular disease, high blood pressure research, clinical cardiology, and epidemiology and prevention. *Circulation*.

[2] Lindner A, Charra B, Sherrard DJ, Scribner BH (1974). Accelerated atherosclerosis in prolonged maintenance hemodialysis. *New England Journal of Medicine*.

[3] Higashi Y, Kihara Y, Noma K (2012). Endothelial dysfunction and hypertension in aging. *Hypertension Research*.

[4] Ross R (1999). Atherosclerosis—an inflammatory disease. *New England Journal of Medicine*.

[5] Locatelli F, Pozzoni P, Tentori F, del Vecchio L (2003). Epidemiology of cardiovascular risk in patients with chronic kidney disease. *Nephrology Dialysis Transplantation*.

[6] Yilmaz MI, Saglam M, Caglar K (2006). The determinants of endothelial dysfunction in CKD: oxidative stress and asymmetric dimethylarginine. *American Journal of Kidney Diseases*.

[7] Kronenberg F (2005). Dyslipidemia and nephrotic syndrome: recent advances. *Journal of Renal Nutrition*.

[8] Chang TI, Desai M, Solomon DH, Winkelmayer WC (2011). Kidney function and long-term medication adherence after myocardial infarction in the elderly. *Clinical Journal of the American Society of Nephrology*.

[9] Japan Atherosclerosis Society (2012). *Japan Atherosclerosis Society Guidelines for Prevention of Atherosclerotic Cardiovascular Diseases 2012*.

[10] Yu M, Kim YJ, Kang DH (2011). Indoxyl sulfate-induced endothelial dysfunction in patients with chronic kidney disease via an induction of oxidative stress. *Clinical Journal of the American Society of Nephrology*.

[11] Krenning G, Dankers PYW, Drouven JW (2009). Endothelial progenitor cell dysfunction in patients with progressive chronic kidney disease. *American Journal of Physiology—Renal Physiology*.

[12] Vanhoutte PM (2009). How we learned to say NO. *Arteriosclerosis, Thrombosis, and Vascular Biology*.

[13] Brodsky SV, Morrishow AM, Dharia N, Gross SS, Goligorsky MS (2001). Glucose scavenging of nitric oxide. *American Journal of Physiology—Renal Physiology*.

[14] Mimura T, Takenaka T, Kanno Y, Moriwaki K, Okada H, Suzuki H (2008). Vascular compliance is secured under angiotensin inhibition in non-diabetic chronic kidney diseases. *Journal of Human Hypertension*.

[15] Verhulst A, D’Haese PC, De Broe ME (2004). Inhibitors of HMG-CoA reductase reduce receptor-mediated endocytosis in human kidney proximal tubular cells. *Journal of the American Society of Nephrology*.

[16] Takenaka T, Seto T, Okayama M (2012). Long-term effects of calcium antagonists on augmentation index in hypertensive patients with chronic kidney disease: a randomized controlled study. *American Journal of Nephrology*.

[17] Kobayashi K, Ohno Y, Takenaka T (2011). Telmisartan lowers home blood pressure and improves insulin resistance without correlation between their changes. *Clinical and Experimental Hypertension*.

[18] Japanese Society of Hypertension (2008). *Guidelines for the Management of Chronic Kidney Diseases; Hypertension*.

[19] Ogihara T, Nakao K, Fukui T (2008). Effects of candesartan compared with amlodipine in hypertensive patients with high cardiovascular risks: candesartan antihypertensive survival evaluation in Japan trial. *Hypertension*.

[20] Suzuki H, Kanno Y, Sugahara S (2008). Effect of angiotensin receptor blockers on cardiovascular events in patients undergoing hemodialysis: an open-label randomized controlled trial. *American Journal of Kidney Diseases*.

[21] Colhoun HM, Betteridge DJ, Durrington PN (2009). Effects of atorvastatin on kidney outcomes and cardiovascular disease in patients with diabetes: an analysis from the Collaborative Atorvastatin Diabetes Study (CARDS). *American Journal of Kidney Diseases*.

[22] Shepherd J, Kastelein JJP, Bittner V (2007). Effect of intensive lipid lowering with atorvastatin on renal function in patients with coronary heart disease: the Treating to New Targets (TNT) study. *Clinical Journal of the American Society of Nephrology*.

[23] Baigent C, Landray MJ, Reith C (2011). The effects of lowering LDL cholesterol with simvastatin plus ezetimibe in patients with chronic kidney disease (Study of Heart and Renal Protection): a randomised placebo-controlled trial. *The Lancet*.

[24] Fellström BC, Jardine AG, Schmieder RE (2009). Rosuvastatin and cardiovascular events in patients undergoing hemodialysis. *New England Journal of Medicine*.

[25] Takenaka T, Takahashi K, Kobayashi T, Oshima E, Iwasaki S, Suzuki H (2002). Oxidized low density lipoprotein (Ox-LDL) as a marker of atherosclerosis in hemodialysis (HD) patients. *Clinical Nephrology*.

[26] Nakamura T, Sato E, Fujiwara N (2010). Atorvastatin reduces proteinuria in non-diabetic chronic kidney disease patients partly via lowering serum levels of advanced glycation end products (AGEs). *Oxidative Medicine and Cellular Longevity*.

[27] Takemoto M, Okabe E, Kobayashi K (2012). Renoprotective effects of atorvastatin om dyslipidemic patients with tyoe2 diabetes: influence on urinary podocyte. *Journal of the Japanese Diabetes Society*.

[28] Mason RP, Walter MF, Day CA, Jacob RF (2005). Intermolecular differences of 3-hydroxy-3-methylglutaryl coenzyme a reductase inhibitors contribute to distinct pharmacologic and pleiotropic actions. *American Journal of Cardiology*.

[29] Siddiqi L, Joles JA, Oey PL, Blankestijn PJ (2011). Atorvastatin reduces sympathetic activity in patients with chronic kidney disease. *Journal of Hypertension*.

[30] Mukerji N, Damodaran TV, Winn MP (2007). TRPC6 and FSGS: the latest TRP channelopathy. *Biochimica et Biophysica Acta*.

[31] Takenaka T, Suzuki H, Okada H (2002). Transient receptor potential channels in rat renal microcirculation: actions of angiotensin II. *Kidney International*.

[32] Giunti S, Calkin AC, Forbes JM (2010). The pleiotropic actions of rosuvastatin confer renal benefits in the diabetic Apo-E knockout mouse. *American Journal of Physiology—Renal Physiology*.

[33] Zwaka TP, Hombach V, Torzewski J (2001). C-reactive protein-mediated low density lipoprotein uptake by macrophages: implications for atherosclerosis. *Circulation*.

[34] Ridker PM, Danielson E, Fonseca FAH (2008). Rosuvastatin to prevent vascular events in men and women with elevated C-reactive protein. *New England Journal of Medicine*.

[35] Schneider MP, Schmidt BM, John S, Schmieder RE (2011). Effects of statin treatment on endothelial function, oxidative stress and inflammation in patients with arterial hypertension and normal cholesterol levels. *Journal of Hypertension*.

[36] Ozaki K, Kubo T, Imaki R (2006). The anti-atherosclerotic effects of lipid lowering with atorvastatin in patients with hypercholesterolemia. *Journal of Atherosclerosis and Thrombosis*.

[37] Epstein M, Campese VM (2005). Pleiotropic effects of 3-hydroxy-3-methylglutaryl coenzyme A reductase inhibitors on renal function. *American Journal of Kidney Diseases*.

